# Enhancement of Curcumin Anti-Inflammatory Effect via Formulation into Myrrh Oil-Based Nanoemulgel

**DOI:** 10.3390/polym13040577

**Published:** 2021-02-14

**Authors:** Wafaa E. Soliman, Tamer M. Shehata, Maged E. Mohamed, Nancy S. Younis, Heba S. Elsewedy

**Affiliations:** 1 Department of Biomedical Sciences, College of Clinical Pharmacy, King Faisal University, Alhofuf, Al-Ahsa 36362, Saudi Arabia; 2 Department of Microbiology and Immunology, Faculty of Pharmacy, Delta University for Science and Technology, Gamasa, Mansoura 11152, Egypt; 3 Department of Pharmaceutics, College of Pharmacy, Zagazig University, Zagazig 44519, Egypt; tshehata@kfu.edu.sa; 4 Department of Pharmaceutical Sciences, College of Clinical Pharmacy, King Faisal University, Alhofuf, Al-Ahsa 36362, Saudi Arabia; Memohamed@kfu.edu.sa (M.E.M.); nyounis@kfu.edu.sa (N.S.Y.); helsewedy@kfu.edu.sa (H.S.E.); 5 Department of Pharmacognosy, College of Pharmacy, Zagazig University, Zagazig 44519, Egypt; 6 Pharmacology Department, Zagazig University, Zagazig 44519, Egypt

**Keywords:** curcumin, nanoemulgel, transdermal, permeation, anti-inflammatory

## Abstract

Background: Curcumin (Cur) possesses a variety of beneficial pharmacological properties including antioxidant, antimicrobial, anti-cancer and anti-inflammatory activities. Nevertheless, the low aqueous solubility and subsequent poor bioavailability greatly limits its effectiveness. Besides, the role of myrrh oil as an essential oil in treating inflammatory disorders has been recently demonstrated. The objective of the current investigation is to enhance Cur efficacy via developing Cur nanoemulgel, which helps to improve its solubility and permeability, for transdermal delivery. Methods: The formulated preparations (Cur gel, emulgel and nanoemulgel) were evaluated for their physical appearance, spreadability, viscosity, particle size, in vitro release and ex vivo drug permeation studies. The in vivo anti-inflammatory activity was estimated using the carrageenan-induced rat hind paw edema method. Results: The formulated Cur-loaded preparations exhibited good physical characteristics that were in the acceptable range of transdermal preparations. The release of Cur from gel, emulgel and nanoemulgel after 12 h was 72.17 ± 3.76, 51.93 ± 3.81 and 62.0 ± 3.9%, respectively. Skin permeation of Cur was significantly (*p* < 0.05) improved when formulated into nanoemulgel since it showed the best steady state transdermal flux (SSTF) value (108.6 ± 3.8 µg/cm^2^·h) with the highest enhancement ratio (ER) (7.1 ± 0.2). In vivo anti-inflammatory studies proved that Cur-loaded nanoemulgel displayed the lowest percent of swelling (26.6% after 12 h). Conclusions: The obtained data confirmed the potential of the nanoemulgel dosage form and established the synergism of myrrh oil and Cur as an advanced anti-inflammatory drug.

## 1. Introduction

Curcumin (Cur) is one of the main constituents of Curcuma longa (turmeric) rhizome, which is a natural compound [1]. It is regarded as the main component responsible for its yellow color [2]. It has demonstrated numerous pharmacological activities, including antioxidant [3], anti-inflammatory [2], antimicrobial [4] and anti-cancer activity [5]. Cur is a lipophilic compound and is practically insoluble in water; further, it possesses poor bioavailability, which seems to be mainly due to its poor absorption, and rapid metabolism and elimination [6,7].

Myrrh oil has been recognized as a natural remedy and is renowned for its substantial effect in treating skin disorders [8]. It comprises numerous components that possess antioxidant and anti-inflammatory effects [9,10]. Likewise, myrrh oil has anticancer [11,12], analgesic [11,13] antibacterial [14], and antithrombin activities [15]. Recently, myrrh oil has been formulated as a nanoemulsion for enhancement of the antihyperlipidemic effect of atorvastatin [16].

Topical anti-inflammatory preparations are designed to be applied directly to the affected area on the skin for pain relief and to treat certain disorders such as inflammation or swelling [17]. In addition, transdermal therapy is regarded as an effective approach that ensures adequate delivery of medicaments with less adverse reactions [18]. It encompasses numerous dosage forms such as a patch, gel, emulgel and nanoemulgel.

Nanoemulsions are considered drug carrier systems that are appropriate for several routes of administration including oral, parenteral and topical delivery. A nanoemulsion is a colloidal dispersion that is thermodynamically stable, formed by two immiscible liquids that are mixed together using proper surfactant to form a single phase [19].

A nanoemulgel is a novel preparation for transdermal delivery that forms as a result of incorporating a nanoemulsion within a hydrogel, which provides the formulation with dual characteristics. It has the ability to deliver hydrophobic drugs, which is considered a great challenge that can limit the drug application [20]. Hydrophobic drugs could be entrapped in the oily phase of the nanoemulsion and then the droplets of the nanoemulsion are incorporated into the gel preparation. This permits a slow release and absorption of the drug upon application [21]. Further, the rheological properties of nanoemulgel are suitable for topical and transdermal drug delivery. Indeed, nanoemulgel is able to avoid the first pass effect, enhance the drug permeation through the skin and provide a controlled and sustained drug delivery [22].

Recently, specific investigations have addressed how to improve Cur solubility. Chowdhury et al., studied an approach to enhance Cur solubility via developing a curcumin–ionic liquid complex [23]. Moreover, Hardiningtyas et al., applied a solid-in-water nanodispersion technique to incorporate Cur and magnify its therapeutic action [24].

Based on these previous studies, a novel approach for developing nanoemulgel intended for anti-inflammatory purposes, incorporating natural products, namely, Cur and myrrh oil is expected to accomplish this aim, with the least side effects. Incorporation of Cur in myrrh oil-based nanoemulsion could intensify the effectiveness and stability of the preparations. To our knowledge, no previous investigations have been carried out in order to enhance Cur solubility and improve skin permeation via a nanoemulgel dosage form prepared by homogenization and ultrasonication techniques. Further, the literature shows no studies on the synergism between Cur and myrrh oil for augmenting the anti-inflammatory effect.

In the current investigation, different transdermal preparations loaded with Cur were developed using myrrh oil in order to enhance the efficacy and the transdermal drug delivery, as well as to reveal the synergistic effect of myrrh oil with Cur. Cur-loaded formulations were assessed for their physical characters, and by in vitro and ex vivo studies. The anti-inflammatory activities and skin irritation studies were evaluated in rats.

## 2. Materials and Methods

### 2.1. Materials

Curcumin, Tween 80, NaCMC and ethanol were purchased from Sigma-Aldrich Co. (St Louis, MO, USA). Propylene glycol (PG) was purchased from Merck (Schuchardh, Hokenbrunn, Germany). Myrrh oil was purchased from NOW^®^ Essential Oils (NOW Foods, Bloomingdale, IL, USA). All other chemicals of analytical grade were purchased from Sigma, USA.

### 2.2. Development of Transdermal Cur Formulations

#### 2.2.1. Development of Gel

Gel base was prepared by sprinkling NaCMC (2% *w*/*w*) into water with constant stirring in a magnetic stirrer (Jeio Tech TM-14SB, Medline Scientific, Oxfordshire, UK) to achieve complete, uniform swelling and get a gel base. A quantified amount of Cur (2% *w*/*w*) was dissolved into ethanol, then vortexed for 5 min. The previous Cur solution was added to the gel base to form a homogenous gel.

#### 2.2.2. Development of Cur-Loaded Emulgel

An accurately weighed amount of Cur (2% *w*/*w*) was dissolved in myrrh oil to form the oily phase. Tween 80 as a surfactant, PG as a co-surfactant and ethyl alcohol as a solvent were added to 20 mL water and vortexed for 5 min to form the aqueous phase. Then, the aqueous phase was added to the oily phase under persistent stirring to get the primary emulsion. On the other hand, the gel base was prepared using the remaining water with gelling agent as mentioned previously. Cur-loaded emulsion was added slowly to the gel base followed by mixing with a mixer (Heidolph RZR 1, Heidolph Instruments, Schwabach, Germany) for 5 min at 3000 rpm until a homogenous emulgel was attained [25].

#### 2.2.3. Development of Nanoemulgel

**Figure 1:**
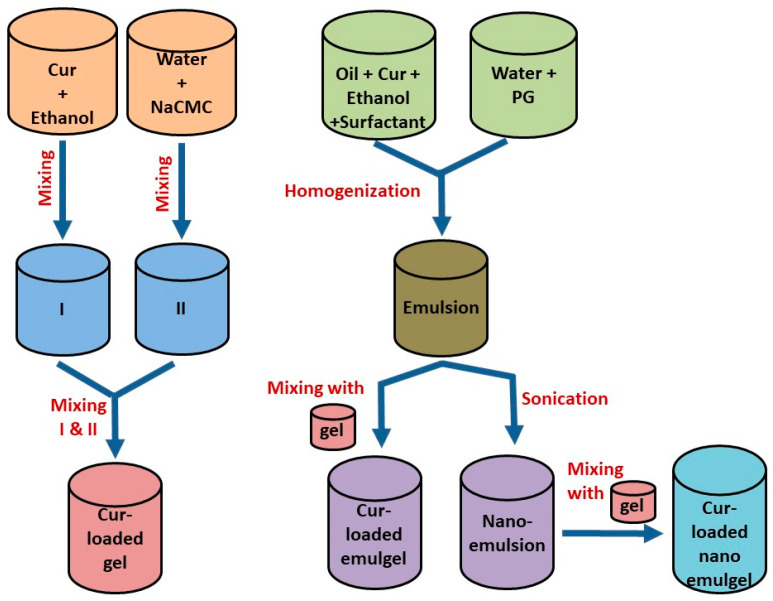
Schematic representation for the development technique of different Cur-loaded formulations.

A similar technique was followed for preparing Cur-loaded emulsion, then it was homogenized for 5 min at 10,000 rpm using a homogenizer (T 25 digital Ultra-Turrax, IKA, Staufen, Germany). Further, it was subjected to sonication for 10 min using a probe sonicator at 150 watt (XL-2000, Qsonica, Newtown, CT, USA) in order to obtain the appropriate nanoemulsion particle size. Cur-loaded nanoemulsion was incorporated into the gel base while mixing for 10 min with a mixer until homogenous cur-loaded nanoemulgel was attained. Table 1 and Figure 1 elucidate the composition and the method used to develop the various transdermal Cur-loaded formulations.

### 2.3. Physical Characterization

#### 2.3.1. Visual Inspection

The formulated transdermal preparations were visually inspected for their color, appearance and homogeneity.

#### 2.3.2. pH Measurement

The pH value of the formulations was detected at room temperature using a calibrated pH meter (MW802, Milwaukee Instruments, Szeged, Hungary).

#### 2.3.3. Spreadability Test

The spreadability of the prepared transdermal formulations was measured using spreadability apparatus, which had a wooden board with a scale and two glass slides. It helps to determine the extent of the area that the formulation could freely spread over the affected part of the skin after being applied. One gram of gel, emulgel or nanoemulgel formulation was placed between two horizontal glass slides (25 cm × 25 cm) and a certain load (500 g) was applied for 1 min. The diameter of the spread of the formulations was measured as it represents the spreadability value [26,27].

#### 2.3.4. Viscosity

A Brookfield viscometer (DV-II + Pro, USA) was employed to determine the viscosity of the formulated gel, emulgel and nanoemulgel using a spindle R5 rotated at 0.5 rpm at 25 ± 0.3 °C [19].

#### 2.3.5. Size and Size Distribution

Particle size and polydispersity (PDI) of Cur-loaded gel, emulgel and nanoemulgel were estimated via measuring their dynamic light scattering using a Zetasizer apparatus (Malvern Instruments Ltd., Worcestershire, UK). The formulations (10 µL) were diluted with 3 mL of distilled water. All the experiments were performed in triplicate [28].

#### 2.3.6. Morphological Evaluation

The morphology of the prepared Cur-loaded nanoemulgel was examined using a scanning electron microscopy (SEM), (JSM-6390LA, JEOL, Tokyo, Japan). The morphology of the formulation was considered at different magnifications (1000 to 95,000). One drop of sample after being diluted was coated with gold under vacuum on metal stubs, and then examined at 5 kv [29].

### 2.4. In Vitro Drug Release Studies

The in vitro release of Cur from the various transdermal formulations and aqueous suspension was investigated using the Agilent fiber optics dissolution system (Agilent Technologies, Santa Clara, CA, USA) according to method previously described by Elsewedy et al. Briefly, 1 g of the formulations was added into glass tubes that were used instead of baskets, and covered with cellophane membrane (MWCO 2000–15,000) from one side. The tubes were maintained in 750 mL of phosphate buffer (pH 7.4) and rotated at 50 rpm at 37 + 0.5 °C. Samples were measured spectrophotometrically at different time intervals (0.25, 0.5, 1, 2 until 6 h) at λ_max_ 425 nm. Each experiment was performed in triplicate [19].

### 2.5. Kinetic Study

In order to assess the correlation coefficient (*r*^2^) and release kinetics of Cur-loaded formulations, the profile of an in vitro drug release study was used. This was achieved by plotting the drug concentration (*Q*) against time (*t*). This study was carried out using various kinetic models as following [30]:

(a) Zero order equation *Q* = *Q*_0_ + *kt*
(b) First order equation *Q* = *Q*_0_ × *ekt*
(c) Higuchi equation *Q* = *k* × *t*_0.5_
(d) Korsmeyer–Peppas equation *Q* = *k* × *t*_n_

where *Q* denotes the quantity released of the drug in time *t*, *Q*_0_ denotes the value of *Q* at time zero, *k* represents the permeation rate constant and *n* signifies the permeation rate exponent. The model that exhibited a linear plot and showed the highest value of (*r*^2^) for the drug release data was considered the best-fit model [31].

### 2.6. Stability Study

Stability studies of the prepared Cur-loaded formulations were carried out to examine their physicochemical properties. The formulations were stored in tightly closed containers and kept at 60% relative humidity, and temperatures of 4 °C and 25 °C over a period of 6 months. The samples were inspected for their physical characteristics and in vitro drug release at the predetermined period of time [32].

### 2.7. Animal Experiment

Male Wistar rats with an average weight of 220 to 250 g were obtained from the Animal Breeding Center, Faculty of Veterinary Medicine, Zagazig University, Egypt and treated according to the Ethical Committee of Animal Handling in the Faculty of Pharmacy, Zagazig University (ZU/FP/282015). The animals were housed in a light and dark cycle in a controlled environment and an ambient temperature with free access to food and water.

### 2.8. Ex Vivo Evaluation (Skin Permeation Study)

#### 2.8.1. Preparation of Rat Skin

Rate skin is regarded as the best choice for implementing a skin permeation study because of its great structural similarity to human skin, it is handled easily and low cost. Using an electric clipper, the dorsal hair skin of male Wistar rats was removed. Afterwards, animals were sacrificed and the skin were excised and the adipose tissue was removed then the skin, hydrated with phosphate buffer (pH 7.4) and stored at 4 °C overnight [33].

#### 2.8.2. Permeation of Cur from Different Transdermal Formulations

An ex vivo permeation study of Cur from all preparations on male Wistar rat skin was implemented using modified Franz diffusion cells developed in our lab [25,34,35]. The skin membranes were mounted in the diffusion cell, with the upside facing the drug-loaded formulation and the dermis facing the receptor media containing 100 mL phosphate buffer (pH 7.4) and 0.02% sodium azide at 37 ± 0.5 °C. Next, 1 g of the formulation (that is equivalent to 1 mg Cur) was applied to the donor area that was attached to the glass tubes and covered with skin membranes. The tubes were suspended into the dissolution apparatus, shielded with Parafilm (Bemis, Oshkosh, WI, USA) to prevent water evaporation and stirred at 100 rpm [33]. Certain parameters correlated with the ex vivo permeation study of Cur across the rat skin were estimated for all formulations including steady state transdermal flux (SSTF) and the enhancement ratio (ER). These parameters were calculated as follow:

SSTF = amount of permeated drug / (area × time);

ER = SSTF from test / SSTF from control.

### 2.9. Anti-Inflammatory Activity

#### Carrageenan-Induced Rat Paw Edema

The anti-inflammatory effect of Cur incorporated in different transdermal preparations was evaluated by the carrageenan-induced rat hind paw edema method. Induction of paw edema in rats was achieved half an hour prior to drug application via subcutaneous injection of 0.5% *w*/*v* carrageenan in saline into the left hind paw where the needle was inserted into the central part of the paw [36]. Thirty rats were randomly divided into six groups, five rats in each as follow:

Group I: Negative control (induced with inflammation without receiving any treatment)

Group II: Positive control (induced with inflammation, treated orally with Cur suspension in water) (10 mg/kg)

Group III: Placebo (induced with inflammation, treated with nanoemulgel without Cur)

Group IV: Treated group (induced with inflammation, treated with Cur-loaded gel)

Group V: Treated group (Induced with inflammation, treated with Cur-loaded emulgel)

Group VI: Treated group (induced with inflammation, treated with Cur-loaded nanoemulgel) [37]

The changes in paw thickness, which represent the inflammatory response, were measured using a digital caliber at specified time intervals of 0, 1, 2, 3, 4, 6 and 12 h. The following Equation (1) presents how to calculate the percentage of swelling [38]:

% of Swelling = ((*V*_t_ − *V*_0_) / *V*_0_) × 100 (1)

where *V*_t_ represents the volume of the carrageenan-treated paw and V_0_ represent the volume of the hind paw at time zero.

### 2.10. Skin Irritation Studies

A skin irritancy test was conducted in male Wistar rats where the hair on the dorsal side of the rat was shaved using clippers one day before starting the experiment. Each formulation was applied on the hair-free skin of rats by spreading it evenly over the tested area. The surface of the skin was monitored and graded for any sensitivity reaction including irritation, edema or erythema (redness) for 7 days following the topical application of the formulation. The sensitivity reaction that was observe was scored as 0, 1, 2, or 3, which signify no reaction, slight erythema, moderate erythema, and severe erythema with or without edema, respectively [22].

### 2.11. Statistical Analysis

Data from treated groups and the control group were compared applying a one-way analysis of variance (ANOVA) followed by the least significant difference (LSD) as a post-hoc test, through SPSS statistics software, version 14 (IBM Corporation, Armonk, NY, USA). Values are expressed as mean ± standard deviation (SD) using at least three independent investigations. The difference was statistically significant at *p* < 0.05.

## 3. Results and Discussion

### 3.1. Physical Characterization

#### 3.1.1. Visual Inspection

Cur-loaded formulations including gel, emulgel and nanoemulgel were effectively developed and assessed for their physical properties as presented in Table 2. The formulations were red, viscous, and shared a homogeneous and smooth appearance.

#### 3.1.2. pH Measurement

Regarding the pH values, these was measured for all developed formulations and the results are displayed in Table 2. pH values ranged between 5.8 ± 0.2 to 6.7 ± 0.3, which are satisfactory and predicted not to cause any skin irritations upon application. The results are similar to those obtained by Mulia et al. who verified the homogeneity of mangosteen nanoemulgel and stated that its pH was from 5.5 to 6.4 [39].

#### 3.1.3. Spreadability Test

Good spreadability is regarded as one of the main standards for transdermal preparations. As displayed in Table 2, the spreadability values of Cur-loaded formulations were 61.2 ± 1.7, 49.2 ± 2.7 and 53.5 ± 2.0 mm for Cur-loaded gel, emulgel and nanoemulgel, respectively. Our results were in accordance with Rajput et al. who estimated the spreadability of their nanogel formulations to be within 53 to 65 mm [40].

#### 3.1.4. Viscosity

Viscosity is considered an essential physical property for transdermal formulations. Subsequently, the viscosity of the developed transdermal formulations was evaluated and the results are shown in Table 2. It was revealed that emulgel (93,300 ± 1053 cP) exhibited significantly higher viscosity than gel and nanoemulgel (57,300 ± 1835 and 79,700 ± 1085 cP) (*p* < 0.05), respectively, which is reasonable and within the proper range. These results are in agreement with Morsy et al., who revealed that the viscosities of different topical atorvastatin formulations ranged between 58,500 and 97,250 cP [25].

#### 3.1.5. Particle Size and PDI

**Figure 2:**
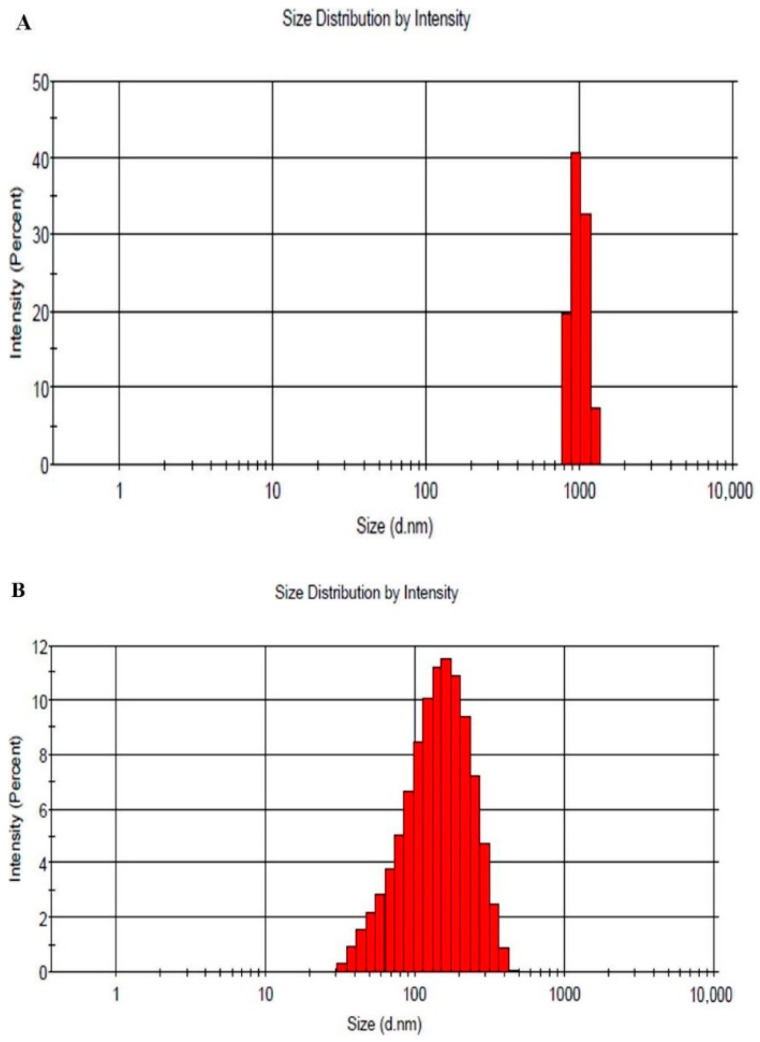
Size distribution of formulated (**A**) cur-loaded emulgel and (**B**) cur-loaded nanoemulgel.

Particle size and PDI of the developed Cur-loaded emulgel and nanoemulgel were assessed and the results are depicted in Figure 2. Emulgel showed a particle size of 1698 nm with PDI 0.338, on the other hand, it was 130 nm with PDI 0.235 for nanoemulgel preparation. Accordingly, the particle sizes of emulgel and nanoemulgel demonstrated a good distribution and fell within a narrow range of sizes. Our findings are in agreement with Ermawati et al., who showed a particle size of 150 nm for the formulation of nanoemulgel gold [41]. Moreover, Dhawan et al., recorded the particle size of piroxicam nanoemulgel to be within the nano size range (125 nm) [42].

#### 3.1.6. Morphological Evaluation

**Figure 3:**
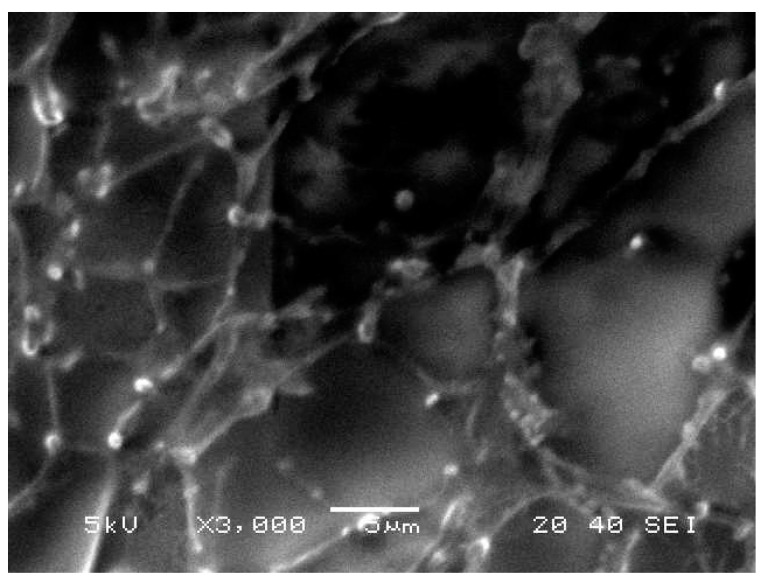
Scanning electron microscope (SEM) of Cur-loaded nanoemulgel.

A demonstrative scanning electron microscopy image of the developed Cur-loaded nanoemulgel is presented in Figure 3. As shown in the figure, small spherical vesicles were scattered through the network of the macromolecular polymer included in the formulation. Additionally, no specific crystals of Cur were apparent in the preparation, suggesting good solubility of the drug.

### 3.2. In Vitro Drug Release Studies

**Figure 4:**
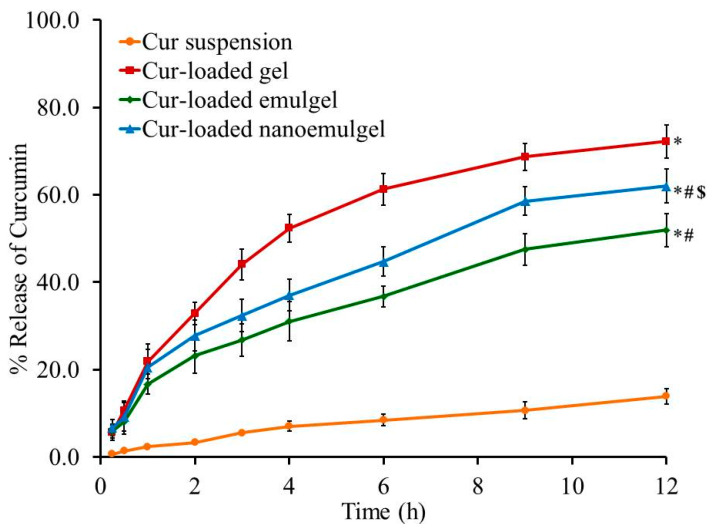
In vitro release study of Cur from different Cur-loaded formulations in phosphate buffer pH 7.4 at 37 °C. Results are expressed as the mean ± SD of three experiments. * *p* < 0.05 compared to Cur suspension. # *p* < 0.05 compared to Cur-loaded gel. $ *p* < 0.05 compared to Cur-loaded emulgel.

The profile of drug release from the developed Cur-loaded formulations and aqueous suspension of Cur in phosphate buffer pH 7.4 was evaluated and the results are shown in Figure 4. The in vitro study signifies that the percentage of Cur released from the preparations was 72.17% ± 3.76%, 51.93% ± 3.81%, and 62.0% ± 3.9% for gel, emulgel and nanoemulgel, respectively, which exceeded the Cur release from the suspension, which was 13.93% ± 1.76% over 12 h. It is obvious that there is a significant difference between the percentage of Cur released from all formulations and that released from the Cur aqueous suspension (*p* < 0.05). Further, a significantly higher percentage of Cur was released from gel compared to that released from emulgel and nanoemulgel (*p* < 0.05), owing to the greater aqueous content incorporated in the gel, which eases the release of the drug into the release media. Alternatively, the higher viscosity of emulgel and nanoemulgel, due to the lower water content and presence of myrrh oil, could lead to slow diffusion of the entrapped drug. This illustrates the significant lower release of Cur from emulgel and nanoemulgel compared to the release from the gel formulation [43]. The higher drug release from the nanoemulgel formulation compared to emulgel could be ascribed to the smaller particle size of nanoemulsion than macroemulsion which provides larger surface area and consequently larger drug release.

### 3.3. Kinetic Study

**Figure 5:**
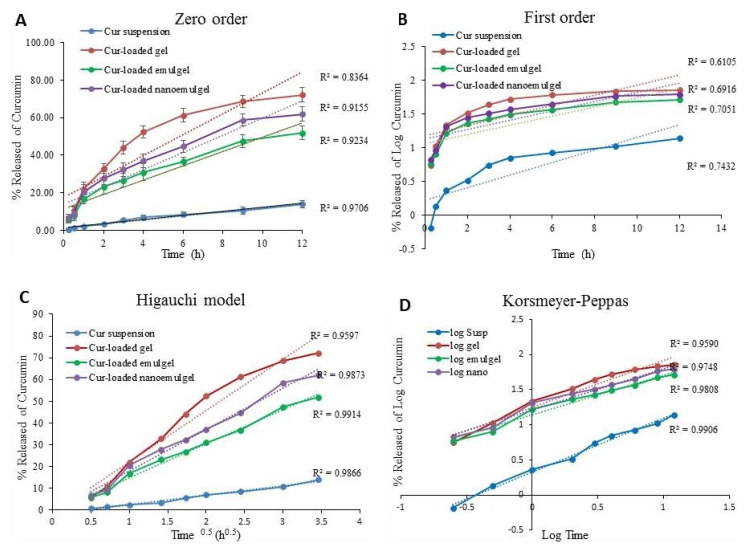
Amount of drug released from Cur-loaded formulations versus curcumin suspension and their kinetics according to (**A**) zero order, (**B**) first order, (**C**) Higuchi model and (**D**) Korsmeyer–Peppas model.

Different kinetic models were employed to describe the release kinetics of Cur from all formulations and the results are presented in Table 3 and Figure 5. The release kinetics were obtained by plotting the amount of drug released versus time and it was apparent that the release kinetics of all Cur-loaded formulations best fitted into the Higuchi diffusion model since it showed a linear plot and demonstrated the highest (*r*^2^) for each kinetic model. The Higuchi model establishes that the drug has released from the matrix type, and perfect sink conditions are attained in the release environment permanently [44,45]. However, the release of Cur from suspension obeyed the Korsmeyer–Peppas model as it achieved a linear plot and the greatest (*r*^2^).

### 3.4. Stability Study

**Figure 6:**
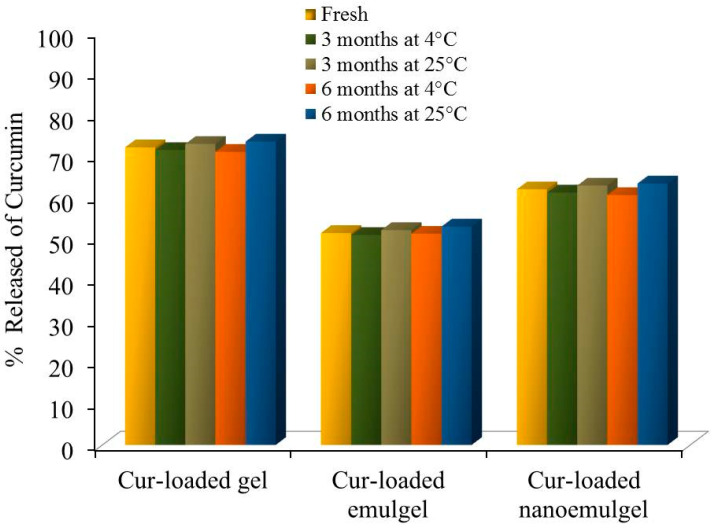
In vitro release study of Cur from different Cur-loaded formulations in phosphate buffer pH 7.4 stored for 3 and 6 months at a relative humidity of 60% and temperature of 4 °C and 25 °C compared with fresh preparations. Results are expressed as the mean ± SD (n = 3).

Stability studies, including physical characteristics and percentage of Cur released from all Cur-loaded formulations, were assessed at 60% relative humidity and temperatures of 4 °C and 25 °C for a period of 3 and 6 months and data are shown in Table 4 and Table 5 and Figure 6. The results exhibited no significant difference in physical properties, namely, the color, homogeneity, pH, spreading and viscosity of stored formulations either at 4 °C or 25 °C when compared to that of fresh preparations. Likewise, no significant changes were identified in the percentage of Cur released from all formulations upon storage compared to their corresponding fresh preparation (*p* < 0.05) (Figure 5). Actually, the gelling agent incorporated into Cur-loaded gel could result in such stability. Our result is similar to that obtained by Mohamed, who determined the essential role of gelling agents in the stability of the preparation [46].

### 3.5. Ex Vivo Evaluation (Skin Permeation Study)

**Figure 7:**
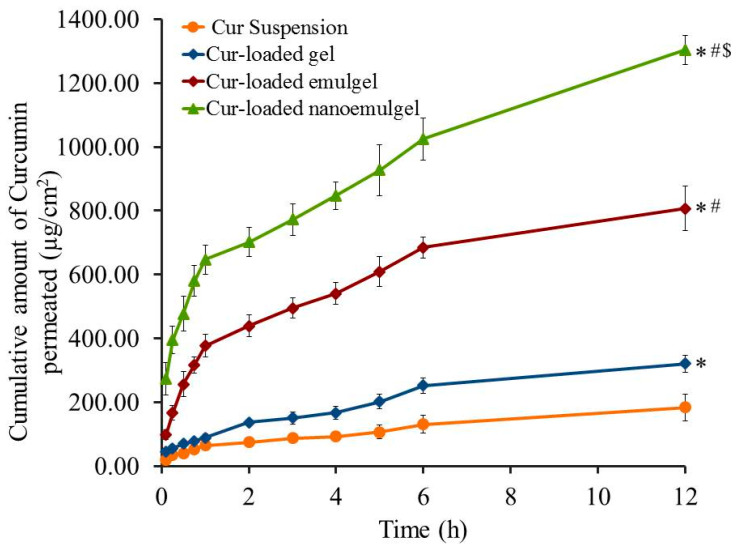
Permeation profiles of Cur across rat skin from different Cur-loaded formulations compared to Cur aqueous suspension (control). Results are expressed as mean ± SD (n = 3). * *p* < 0.05 compared to Cur aqueous suspension. # *p* < 0.05 compared to Cur-loaded gel. $ *p* < 0.05 compared to Cur-loaded emulgel.

The permeation of drug through biological membranes is greatly affected by several factors, the most essential of these is the route of transport selected for the drug permeation [47]. Permeability of Cur through rat skin from different developed Cur-loaded formulations was estimated and compared to permeation from Cur suspension and the results are presented in Figure 7. It is obvious that Cur permeated from Cur-loaded nanoemulgel through rat skin is significantly higher than that permeated from Cur-loaded gel, emulgel and Cur aqueous suspension (*p* < 0.05). It is apparent from Table 6 that SSTF of Cur aqueous suspension exhibits a significantly lower value than other Cur-loaded formulations. Nevertheless, the drug permeability of Cur-loaded gel is enhanced 1.7 ± 0.2 times with a SSTF of 26.7 ± 2.3 µg/cm^2^·h, which can be ascribed to the colloidal nature of gel preparation [48]. Remarkably, Cur-loaded nanoemulgel significantly improved the skin permeation of the drug (*p* < 0.05), hence it exhibited the greatest SSTF value (108.6 ± 3.8 µg/cm^2^·h) with the highest ER (7.1 ± 0.2) compared to all formulations under investigation. It is highly noticeable that incorporating surfactant and penetration enhancer in emulgel and nanoemulgel formulations can boost Cur flux. Further, the water in the external phase can result in hydrating the stratum corneum, and accordingly, it enhances swelling of the cell and facilitates the drug passage [49]. Our findings were in accordance with Thomas et al., who found that the skin permeation of Cur for different nanoemulsion preparations ranged from 75.3 µg/cm^2^·h to 107.1 µg/cm^2^·h [50]. Overall, the small particle size of the developed Cur-loaded nanoemulgel provides a larger surface area that eases the permeation of the drug and allows a higher concentration of drug to be released in the treated area [51]. Morsy et al.’s findings are in agreement with our results since a significantly higher permeation of atorvastatin from nanoemulgel through rat skin was detected compared to emulgel, gel and atorvastatin solution [25].

### 3.6. Anti-Inflammatory Activity

#### Carrageenan-Induced Rat Paw Edema

**Figure 8:**
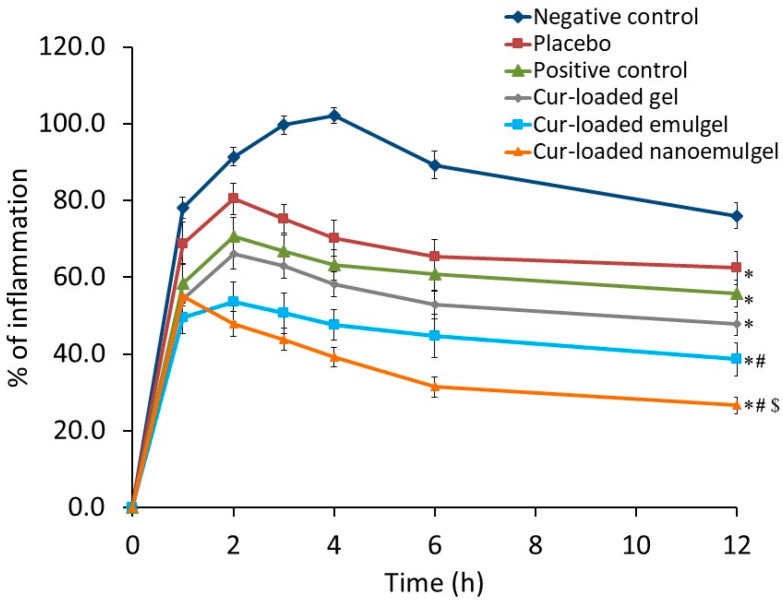
Effects of Cur-loaded formulations on carrageenan-induced paw edema in rats. Results are expressed as mean with the bar showing SD (n = 5). * *p* < 0.05 compared to negative control treated group. # *p* < 0.05 compared to Cur-loaded gel treated group. $ *p* < 0.05 compared to Cur-loaded emulgel treated group.

The anti-inflammatory activity of Cur-loaded formulations on carrageenan-induced rat hind paw edema was evaluated and compared to negative control (non-treated group), positive control (treated orally with Cur suspension) and placebo (treated with nanoemulgel without Cur) and result is displayed in Figure 8. A significant increase (*p* < 0.05) in the inflammation of the negative control group was observed following carrageenan injection, which was achieved after 4 h (102.2%) compared to the non-inflamed rat hind paw. However, significantly lower inflammation was exhibited by the positive control treated animals and placebo group, hence, maximum inflammation (70.6 and 80.4%) was achieved 2 h after carrageenan injection (*p* < 0.05).

On the other side, a significantly lower percentage of inflammation was attained in animals treated with Cur-loaded formulations when compared with other groups under investigation (*p* < 0.05). However, no significant difference was detected between group treated with Cur-loaded gel and positive control group or placebo group (*p* < 0.05). Interestingly, the Cur-loaded nanoemulgel treated group demonstrated the highest percentage of inflammation 1 h following the treatment, subsequently, there was a significant decrease in the percentage of inflammation compared with other treated groups, which achieved 26.6%, 12 h after the transdermal application of the preparation (*p* < 0.05). The nano size of the initial nanoemulsion and the great permeation of the developed nanoemulgel across the rat skin could play a major role in our findings [52]. The results are similar to those obtained by Astuti et al. who stated that mangosteen nanoemulgel provides a highest percentage of edema inhibition (*p* < 0.05) than mangosteen gel [53]. The effects of the placebo treated group in decreasing the inflammation is noteworthy as it reached 62.4% after 12 h, which verifies the role of myrrh oil in lowering the inflammation, as mentioned previously by Su et al. who detected a significant reduction in inflammation for groups treated with myrrh extract [54]. In a nutshell, the significant reduction in inflammation of animals treated with Cur-loaded nanoemulgel proved the synergistic effect of both Cur and myrrh oil.

### 3.7. Skin Irritation Study

All formulations under investigation revealed a score of 0 on the sensitivity reaction scale following their application on the skin of the rats’ backs. The area supplied with the tested formulation was observed for 3 days and no irritation, edema or erythema was detected during the entire period of study. The results are in accordance with Mao et al., who observed no irritation on skin treated with eprinomectin nanoemulgel [20].

## 4. Conclusions

Cur-loaded formulations were prepared and our investigation showed that they exhibited good physical properties with a relevant anti-inflammatory effect. Further, the data elucidated that myrrh oil considerably boosted the in vivo activity of Cur-loaded formulations, especially Cur-loaded nanoemulgel, which confirms the synergism between Cur and myrrh oil. It can be concluded that the nanoemulgel formulation could be considered as a potential vehicle for transdermal delivery of Cur.

## Figures and Tables

**Table 1:** Composition of various Cur-loaded topical preparations.

Formulation	Cur (g)	NaCMC (g)	Myrrh Oil (mL)	Ethanol (g)	PG (g)	Tween 80 (mL)	Water to (g)
Cur-loaded gel	1	1	-	-	-	1	50
Cur-loaded emulgel	1	1	5	2	1	1	50
Cur-loaded nanoemulgel	1	1	5	2	1	1	50

**Table 2:** Physical characterization of Cur-loaded formulations.

Properties	Cur-Loaded Gel	Cur-Loaded Emulgel	Cur-Loaded Nanoemulgel
Color and homogeneity	Reddish homogenous	Reddish homogenous	Yellow homogenous
pH	5.8 ± 0.2	6.7 ± 0.3	6.1 ± 0.2
Spreadability (mm)	61.2 ± 1.7	49.2 ± 2.7 *	53.5 ± 2.0 *^,#^
Viscosity (cP)	57,300 ± 1835	93,300 ± 1053 *	79,700 ± 1085 *^, #^

Values are expressed as mean ± (SD) using one-way ANOVA. * *p* < 0.05 compared to Cur-loaded gel. # *p* < 0.05 compared to Cur-loaded emulgel.

**Table 3:** Release kinetics of different Cur-loaded formulations.

Kinetic Model	Cur Suspension	Cur-Loaded Gel	Cur-Loaded Emulgel	Cur-Loaded Nanoemulgel
Zero order model	0.9706	0.8364	0.9234	0.9155
First order model	0.7432	0.6105	0.7051	0.6916
Higuchi model	0.9866	0.9597	0.9914	0.9873
Korsmeyer–Peppas	0.9906	0.9590	0.9808	0.9748

**Table 4:** Physical characterization of Cur-loaded formulations following 3 months storage at a relative humidity of 60% and temperature of 4 °C.

Properties	Temperature	Cur-Loaded Gel	Cur-Loaded Emulgel	Cur-Loaded Nanoemulgel
**Color and homogeneity**	4 °C	Reddish homogenous	Reddish homogenous	Reddish homogenous
	25 °C	Reddish homogenous	Reddish homogenous	Reddish homogenous
**pH**	4 °C	6.0± 0.3	6.9 ± 0.2	6.2 ± 0.2
	25 °C	5.9 ± 0.4	6.9 ± 0.1	6.2 ± 0.3
**Spreadability (mm)**	4 °C	60.1 ± 1.1	48.4 ± 1.3 *	52.2 ± 1.2 *^, #^
	25 °C	61.7 ± 1.3	50.0 ± 2.3 *	53.8 ± 1.5 *^, #^
**Viscosity (cP)**	4 °C	58,200 ± 950	94,200 ± 884 *	80,200 ± 965 *^, #^
	25 °C	56,030 ± 1680	92,100 ± 1025 *	78,300 ±926 *^, #^

Values are expressed as mean ± (SD). * *p* < 0.05 compared to BRU-loaded gel. # *p* < 0.05 compared to BRU-loaded emulgel.

**Table 5:** Physical characterization of Cur-loaded formulations following 6 months storage at a relative humidity of 60% and temperature of 4 °C.

Properties	Temperature	Cur-Loaded Gel	Cur-Loaded Emulgel	Cur-Loaded Nanoemulgel
**Color and homogeneity**	4 °C	Reddish homogenous	Reddish homogenous	Reddish homogenous
	25 °C	Reddish homogenous	Reddish homogenous	Reddish homogenous
**pH**	4 °C	5.9 ± 0.2	6.9 ± 0.4	6.3 ± 0.3
	25 °C	6.0 ± 0.5	6.9 ± 0.3	6.4 ± 0.2
**Spreadability (mm)**	4 °C	59.4 ± 1.3	46.5 ± 1.4 *	51.1 ± 1.5 *^, #^
	25 °C	62.5 ± 2.1	50.7 ± 2.3 *	55.0 ± 1.9 *^, #^
**Viscosity (cP)**	4 °C	59,300 ± 2025	95,300 ± 1125 *	81,200 ± 884 *^, #^
	25 °C	55,150 ± 1738	91,200 ± 1033 *	77,200 ±1779 *^, #^

Values are expressed as mean ± (SD). * *p* < 0.05 compared to BRU-loaded gel. # *p* < 0.05 compared to BRU-loaded emulgel.

**Table 6:** Ex vivo skin permeation parameters (SSTF and ER) of different Cur-loaded formulations.

Formula	SSTF µg/cm^2^·h	ER
Cur aqueous suspension	15.3 ± 3.5	1
Cur-loaded gel	26.7 ± 2.3 *	1.7 ± 0.2 *
Cur-loaded emulgel	67.2 ± 5.9 *^,#^	4.4 ± 0.4 *^,#^
Cur-loaded nanoemulgel	108.6 ± 3.8 *^,#,^^■^	7.1 ± 0.2 *^,#,^^■^

Values are expressed as mean ± SD. * *p* < 0.05 compared to Cur aqueous suspension. # *p* < 0.05 compared to Cur-loaded gel. ■ *p* < 0.05 compared to Cur-loaded emulgel.

## Data Availability

Not applicable.
